# Super-resolution deep learning reconstruction to evaluate lumbar spinal stenosis status on magnetic resonance myelography

**DOI:** 10.1007/s11604-025-01787-5

**Published:** 2025-04-23

**Authors:** Koichiro Yasaka, Yusuke Asari, Yuichi Morita, Mariko Kurokawa, Taku Tajima, Hiroyuki Akai, Naoki Yoshioka, Masaaki Akahane, Kuni Ohtomo, Osamu Abe, Shigeru Kiryu

**Affiliations:** 1 Department of Radiology, Graduate School of Medicine, The University of Tokyo, 7-3-1 Hongo, Bunkyo-Ku, Tokyo 113-8655 Japan; 2 Department of Radiology, International University of Health and Welfare Narita Hospital, 852 Hatakeda, Narita, Chiba 286-0124 Japan; 3 Department of Radiology, International University of Health and Welfare Mita Hospital, 1-4-3 Mita, Minato-Ku, Tokyo 108-8329 Japan; 4 Department of Radiology, The Institute of Medical Science, The University of Tokyo, 4-6-1 Shirokanedai, Minato-Ku, Tokyo 108-8639 Japan; 5 International University of Health and Welfare, 2600-1 Ktiakanemaru, Ohtawara, Tochigi 324-8501 Japan

**Keywords:** Lumbar spinal stenosis, MRI, Deep learning, Reconstruction, Super-resolution

## Abstract

**Purpose:**

To investigate whether super-resolution deep learning reconstruction (SR-DLR) of MR myelography-aided evaluations of lumbar spinal stenosis.

**Material and methods:**

In this retrospective study, lumbar MR myelography of 40 patients (16 males and 24 females; mean age, 59.4 ± 31.8 years) were analyzed. Using the MR imaging data, MR myelography was separately reconstructed via SR-DLR, deep learning reconstruction (DLR), and conventional zero-filling interpolation (ZIP). Three radiologists, blinded to patient background data and MR reconstruction information, independently evaluated the image sets in terms of the following items: the numbers of levels affected by lumbar spinal stenosis; and cauda equina depiction, sharpness, noise, artifacts, and overall image quality.

**Results:**

The median interobserver agreement in terms of the numbers of lumbar spinal stenosis levels were 0.819, 0.735, and 0.729 for SR-DLR, DLR, and ZIP images, respectively. The imaging quality of the cauda equina, and image sharpness, noise, and overall quality on SR-DLR images were significantly better than those on DLR and ZIP images, as rated by all readers (*p* < 0.001, Wilcoxon signed-rank test). No significant differences were observed for artifacts on SR-DLR against DLR and ZIP.

**Conclusions:**

SR-DLR improved the image quality of lumbar MR myelographs compared to DLR and ZIP, and was associated with better interobserver agreement during assessment of lumbar spinal stenosis status.

## Introduction

Lumbar spinal stenosis is a relatively common disease that may be painful, especially in older patients [1]. Annually, 103 million persons develop such stenosis worldwide [2]. Imaging is useful when seeking to confirm a structural diagnosis. Magnetic resonance (MR) myelography, introduced in 1992, reveals the contents of the spinal canal as well as does conventional myelography, but in a noninvasive manner [3]. Song et al. found that MR myelography was associated with better interobserver agreement during assessment of lumbar spinal stenosis than was evaluation of conventional MR images [4]. While MR myelography can be imaged with 3D sequences, 2D MR myelography can be performed within a shorter time. Considering that many patients who undergo lumbar MR examination tend to have difficulties in maintaining the same posture for a long time, shorter imaging time of 2D MR myelography would have a strong merit over 3D sequences.

The cauda equina and filum terminale within the lumbar spinal canal are both tiny structures [5, 6]. Therefore, if these are to be viewed, images of high spatial resolution are essential. In this context, zero-filling interpolation (ZIP) that improves the spatial resolution of MR images subjected to reconstruction [7] is commonly used in daily clinical practice [8]. However, some limitations are apparent; ZIP exhibits artifacts when the interpolation factors employed are high [9].

Recently, deep learning applications have attracted considerable attention on the part of radiologists [10–12]. Deep learning not only supports diagnoses afforded by imaging [13–21] but also that of image-processing including deep learning reconstruction (DLR), which has been shown to reduce noise included in images [22–28]. Very recently, super-resolution deep learning reconstruction (SR-DLR), a deep learning-based image-processing algorithm, has become available. SR-DLR not only reduces image noise but also enhances the spatial resolution afforded by ZIP by employing a high interpolation factor that does not generate significant artifacts. This is possible because the neural network is specifically trained to reduce artifacts associated with higher interpolation factors [29]. Therefore, we hypothesized that SR-DLR would enhance depiction of the cauda equina and filum terminale, thus further improving interobserver agreement during assessment of lumbar spinal stenosis apparent on MR myelographs.

The aim of this study was to investigate the utility of SR-DLR in terms of evaluation of lumbar spinal stenosis on 2D MR myelographs, as compared to conventional DLR and ZIP images.

## Materials and methods

This retrospective study was approved by our Institutional Review Board, which waived the requirement for written, informed patient consent given the retrospective nature of the work.

### Patients

Patients who underwent lumbar MR examinations including 2D myelography from November 2023 to February 2024 were included (*n* = 45). The patients who were status after lumbar surgery were excluded (*n* = 5). Thus, 40 patients (16 males and 24 females; mean age, 59.4 ± 31.8 years; mean height, 1.61 ± 0.06 m; mean weight, 60.1 ± 10.7 kg) were included in the final analysis.

### MR imaging

All patients underwent MR imaging using a 3-T MR unit (Vantage Centurian [Canon Medical Systems, Otawara, Japan]). The MR imaging parameters for 2D myelography using the fast advanced spin echo sequence were: repetition time, 2000 ms; echo time, 750 ms; flip angle, 90º; number of acquisitions, 2; number of phase-encoding steps, 128; echo train length, 214; and pixel bandwidth, 651 Hz. Using the MR imaging data, three types of images (i.e., SR-DLR [Precise IQ Engine; Canon Medical Systems] images, DLR [Advanced intelligent Clear-IQ Engine; Canon Medical Systems] images, and ZIP images) were reconstructed. The reconstruction parameters were reconstruction diameter, 330.75 mm for SR-DLR, DLR, and ZIP images; slice thickness, 55 mm for SR-DLR, DLR, and ZIP images; zero- filling interpolation factor, 3 for SR-DLR, 1 for DLR, and 2 for ZIP images; rows/columns, 1,056/1,056 for SR-DLR, 352/352 for DLR, and 704/704 for DLR and ZIP images; and pixel spacing, 0.3125 mm for SR-DLR, 0.9375 mm for DLR, and 0.4688 mm for DLR and ZIP images.

### The SR-DLR algorithm

SR-DLR reduces artifacts by using high interpolation factors; image noise also falls. This is achieved by employing two neural networks [29–32]. When using SR-DLR, image noise is first reduced by a neural network trained using low-quality data and high-quality MR images as the inputs and the references, respectively. The output images are processed using the fast Fourier transform followed by zero-filling interpolation of the k-space with a factor of 3. Subsequently, the data are processed employing the inverse fast Fourier transform and input to a second neural network trained to reduce artifacts caused when ZIP analysis features high interpolation factors [29].

### Image analyses

Three radiologists (readers 1/2/3 with 7/3/2 years of imaging experience, respectively) analyzed all images. A radiologist with 14 years of imaging experience randomized all image sets prior to analyses. All three radiologists were blinded to patient background data and MR imaging information. They independently evaluated all image sets in terms of:

* The number of levels in which lumbar spinal stenosis was apparent under the conus medullaris (lumbar spinal stenosis was defined as the complete obscuration of cerebrospinal fluid, which indicates the entire cauda equina as a bundle, as in [33, 34]),

* The resolutions of the cauda equina and filum terminale graded using a 4-point scale (4 = clear, 3 = slightly unclear in only a small region, 2 = unclear in some regions, 1 = unclear) (in this evaluation, levels that exhibited lumbar spinal stenosis were excluded),

* Sharpness graded using a 4-point scale (4 = sharp, 3 = slightly blurred but only in a small region, 2 = blurred in some regions, 1 = blurred to an extent that diagnosis was impossible),

* Noise assessed using a 4-point scale (4 = almost no noise, 3 = slight noise, 2 = rather prominent noise, 1 = severe noise that rendered diagnosis impossible),

* Artifacts evaluated employing a 3-point scale (3 = almost no artifacts, 2 = a few artifacts, 1 = severe artifacts that rendered diagnosis impossible),

* Overall quality measured using a 4-point scale (4 = adequate quality in terms of diagnosis, 3 = room for improvement in terms of image quality, 2 = difficulties encountered during diagnosis, 1 = totally unacceptable image quality).

The image sets of five patients who were excluded from the principal analyses were used for training before the analyses of other patients, to allow the three readers to familiarize themselves with the evaluation algorithms.

### Quantitative image analysis

A radiologist with 14 years of imaging experience placed a region of interest on the spinal cord and measured the average and standard deviation of its signal intensity.

### Statistical analyses

Statistical analyses were performed using R version 4.1.2 (https://www.r-project.org/). The extent of interobserver agreement in terms of evaluation of lumbar spinal stenosis and the number of levels of such stenosis were calculated using Cohen’s kappa analysis and Cohen’s weighted kappa analysis with quadratic weighting, respectively. Image quality scores were compared between SR-DLR *vs.* DLR and SR-DLR *vs.* ZIP employing the Wilcoxon signed-rank test. Because of the multiple comparisons, the Bonferroni correction was applied. Therefore, *P* < 0.025 (= 0.050/2) was considered to indicate a statistically significant difference.

## Results

### Interobserver agreement in terms of evaluation of lumbar spinal stenosis

**Fig. 1:**
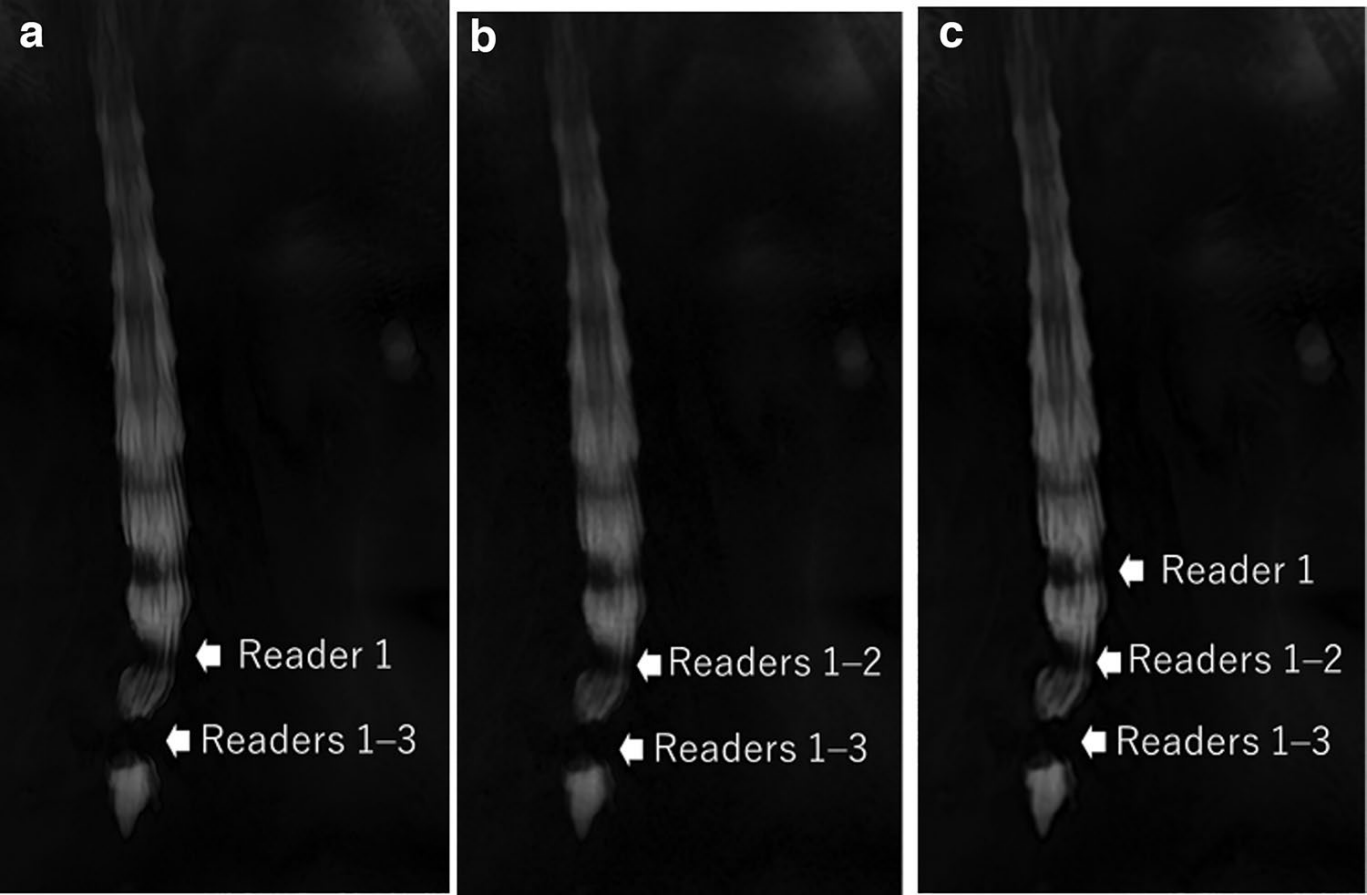
A lumbar MR myelograph of an 80-year-old female patient. Readers 1/2/3 rated the number of levels with lumbar spinal stenosis status as 2/1/1 on the SR-DLR image (**a**), 3/2/1 on the DLR image (**b**), and as 2/2/1 on the ZIP image (**c**). Arrows show the stenotic sites which were indicated by each reader after the completion of all evaluations

**Fig. 2:**
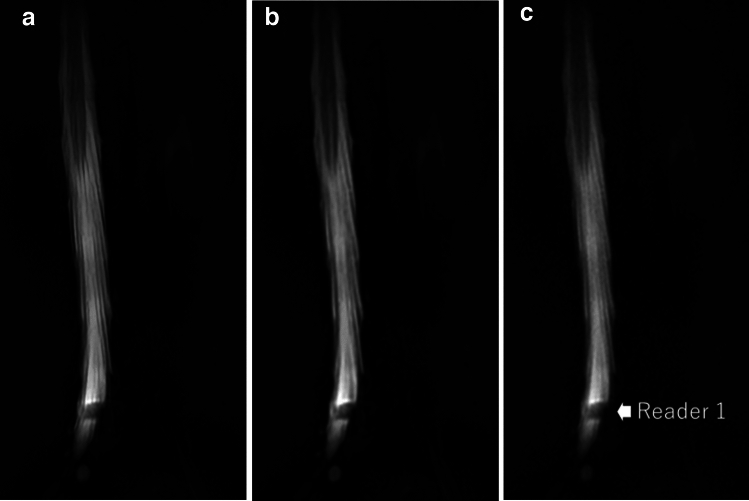
A lumbar MR myelograph of a 23-year-old female patient. The readers 1/2/3 rated the number of levels with lumbar spinal stenosis status as 0/0/0 on the SR-DLR image (**a**), 0/0/0 on the DLR image (**b**), and as 1/0/0 on the ZIP image (**c**). An arrow shows the stenotic site which was indicated by each reader after the completion of all evaluations

Representative patient images are shown in Figs. 1 and 2. The average numbers of levels in which lumbar spinal stenosis was present per patient as evaluated by readers 1/2/3 were 0.70/0.55/0.40, 0.68/0.63/0.43, and 0.70/0.60/0.40 on the SR-DLR, DLR, and ZIP images, respectively. The median interobserver agreements in terms of the numbers of levels of lumbar spinal stenosis were 0.819, 0.735, and 0.729 for the SR-DLR, DLR, and ZIP images, respectively.

### Image quality assessment

**Fig. 3:**
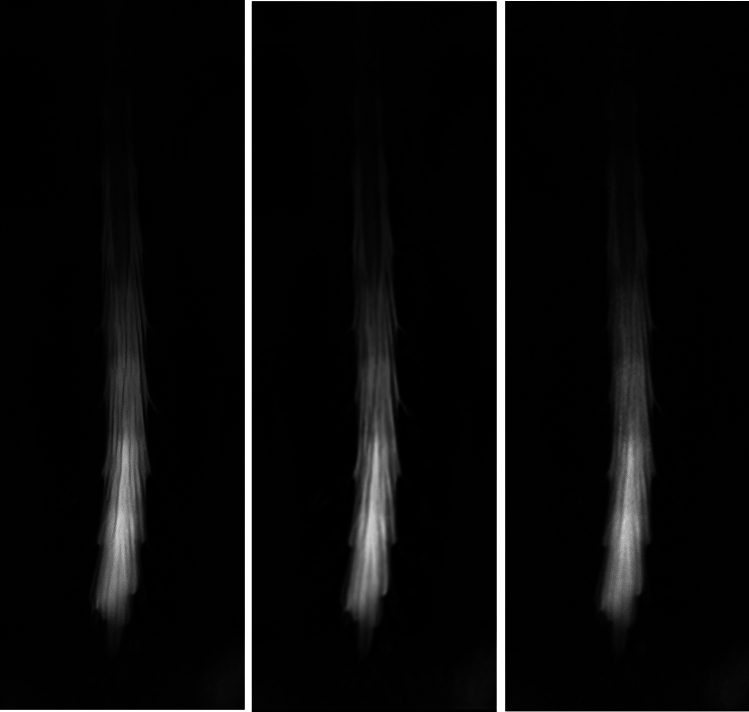
A lumbar MR myelograph of a 24-year-old male patient. Readers 1/2/3 rated the clarity of the cauda equina and filum terminale, sharpness, and noise, as 4 (clear)/3 (slightly unclear)/4, 4 (sharp)/4/4, and 4 (almost no noise)/4/3 (slight noise) respectively in the SR-DLR image (**a**); 4/3/3, 4/3 (slightly blurred)/3, and 4/3/3 respectively, in the DLR image (**b**); and as 2 (unclear in some regions)/3/3, 2 (blurred in some regions)/3/3, and 3/3/3 respectively, in the ZIP image (**b**)

The quality scores for the SR-DLR, DLR, and ZIP images are summarized in Table 1. The cauda equina and filum terminale depictions, sharpness, noise, and overall image quality of SR-DLR images were rated as significantly better than those of DLR and ZIP images by all readers (*p* < 0.001) (Fig. 3). No significant differences were observed for artifacts between SR-DLR *vs.* DLR and SR-DLR *vs.* ZIP by all readers (*p* ≥ 0.037).

**Table 1:** Image quality scores

	Number of patients for each score			Comparison	
	SR-DLR	DLR	ZIP	SR-DLR vs. DLR	SR-DLR vs. ZIP
Depiction of cauda equina and filum terminale (4/3/2/1)					
Reader 1	25/11/4/0	6/20/12/2	1/12/21/6	< 0.001*	< 0.001*
Reader 2	25/15/0/0	1/29/10/0	1/31/7/1	< 0.001*	< 0.001*
Reader 3	23/17/0/0	0/23/17/0	0/19/21/0	< 0.001*	< 0.001*
Sharpness (4/3/2/1)					
Reader 1	26/12/2/0	6/18/14/2	1/10/22/7	< 0.001*	< 0.001*
Reader 2	25/15/0/0	0/15/25/0	1/27/12/0	< 0.001*	< 0.001*
Reader 3	26/12/2/0	1/29/10/0	0/21/19/0	< 0.001*	< 0.001*
Noise (4/3/2/1)					
Reader 1	18/21/1/0	7/22/10/1	0/11/26/3	< 0.001*	< 0.001*
Reader 2	38/2/0/0	0/15/25/0	7/32/1/0	< 0.001*	< 0.001*
Reader 3	25/15/0/0	3/28/9/0	1/24/15/0	< 0.001*	< 0.001*
Artifact (3/2/1)					
Reader 1	39/1/0	37/3/0	34/6/0	0.037	0.346
Reader 2	39/1/0	40/0/0	39/1/0	1.000	1.000
Reader 3	39/1/0	39/1/0	39/1/0	1.000	1.000
Overall image quality (4/3/2/1)					
Reader 1	22/16/2/0	6/22/11/1	1/11/22/6	< 0.001*	< 0.001*
Reader 2	28/11/1/0	0/24/16/0	1/31/7/1	< 0.001*	< 0.001*
Reader 3	28/12/0/0	1/26/13/0	0/24/16/0	< 0.001*	< 0.001*

*DLR* deep learning reconstruction, *SR-DLR* super-resolution deep learning reconstruction, *ZIP* zero-filling interpolation

The numbers of patients, with the various scores (higher scores indicate better quality), are shown. Comparisons employed the Wilcoxon signed-rank test

^*^Statistically significant difference

### Quantitative image analysis

There were no statistically significant differences in the signal-to-noise ratio of the spinal cord in SR-DLR (18.0 ± 10.6) compared to that in DLR (16.8 ± 10.0, *p* = 0.301) and ZIP (17.1 ± 7.8, *p* = 0.305).

## Discussion

In this study, we found that SR-DLR improved interobserver agreement on evaluations of lumbar spinal stenosis as revealed by 2D MR myelographs. We also found that the cauda equina and filum terminale depictions, sharpness, noise, and overall image quality were significantly better on SR-DLR than conventional DLR and ZIP images.

It has been reported that MR myelography (kappa values = 0.771–0.782) was superior to conventional MR imaging (kappa values = 0.649–0.727) in terms of interobserver agreement during evaluation of lumbar spinal stenosis [4]. In our study, we found that SR-DLR images (kappa values = 0.819) further improved interobserver agreement during evaluations of lumbar spinal stenosis compared to conventional DLR and ZIP images (kappa values = 0.729–0.735). The use of 2D MR myelographs reconstructed via SR-DLR will enhance the diagnostic confidence of radiologists who assess lumbar MR images in terms of degenerative change.

Our study showed that image quality, thus sharpness and image noise, of SR-DLR images was significantly better than that of conventional DLR and ZIP images. In the past, in efforts to improve the sharpness of MR images, smaller voxel sizes and low ZIP interpolation factors have been commonly employed. However, a small voxel size is known to be associated with increased noise. SR-DLR is a new strategy that improves the sharpness of an MR image without increasing noise or artifacts [29]. SR-DLR improves spatial resolution using a ZIP technique with a high interpolation factor. The size of the filum terminale in the caudal part of the dural sac was reported to be 0.69 mm in a previous cadaver study [6]. In our study, SR-DLR was associated with a smaller pixel size (0.3125 mm), compared to that of conventional ZIP (0.4688 mm), which would effectively improve depictions of the cauda equina and filum terminale. On the contrary, use of a high interpolation factor has been known to cause artifacts. To circumvent this issue, SR-DLR uses a neural network trained to reduce artifacts. In fact, the artifacts were not increased in SR-DLR compared to conventional DLR and ZIP images in our study. In addition, although the conventional strategy used to improve spatial resolution increases image noise, SR-DLR in fact reduces image noise by using a neural network trained employing low-quality images, and high-quality images with minimal noise, as the input and reference data, respectively. All readers agreed that image noise was significantly reduced in SR-DLR compared not only to ZIP images but also to DLR images.

Our work had certain limitations. First, the extent of lumbar spinal stenosis apparent on MR myelographs and patient symptoms were not correlated. Second, the study was retrospective in nature. Although the results are promising, a future prospective study is required to consolidate the findings. Third, because it was hard to find homogeneous regions within the cerebrospinal fluid allowing to place regions of interest, only the signal-to-noise ratio for the spinal cord was evaluated in the quantitative image analyses. However, the qualitative image analyses, which are clinically more relevant, successfully showed superiority of SR-DLR over DLR and ZIP. Fourth, because it was hard to identify the level where spinal stenosis was observed with 2D lumbar MR myelographs, we did not evaluate the concordance rate of the location of spinal stenosis. However, previous studies have reported that multiple levels of vertebral lesions are associated with the presence of symptoms from lumbar spinal stenosis [35, 36]. Therefore, the assessment of the number of stenotic sites would have clinical impact. Fifth, we evaluated the impact of SR-DLR only on 2D myelography. How this technology affects the evaluation of lumbar spinal stenosis using 3D T2-weighted images, T2-weighted axial images, or T2-weighted sagittal images needs to be investigated in future studies. Finally, the detailed SR-DLR algorithms differ slightly across the various vendors. Therefore, the results of our study would not necessarily be applicable to similar algorithms from other vendors.

In conclusion, SR-DLR improved the image quality of 2D lumbar MR myelographs compared to conventional DLR and ZIP and was also associated with improved interobserver agreement during evaluations of lumbar spinal stenosis.
